# Maize *ZmRACK1* Is Involved in the Plant Response to Fungal Phytopathogens

**DOI:** 10.3390/ijms15069343

**Published:** 2014-05-26

**Authors:** Baosheng Wang, Jingjuan Yu, Dengyun Zhu, Yujie Chang, Qian Zhao

**Affiliations:** 1 State Key Laboratory of Agribiotechnology, College of Biological Sciences, China Agricultural University, Beijing 100193, China; E-Mails: yujj@cau.edu.cn (J.Y.); zhudy@cau.edu.cn (D.Z.); changyujie@cau.edu.cn (Y.C.); 2 Inner Mongolia Academy of Agriculture and Animal Husbandry Sciences, Hohhot 010031, China; E-Mail: cauwbs@gmail.com

**Keywords:** *Zea mays*, RACK1, disease resistance

## Abstract

The receptor for activated C kinase 1 (RACK1) belongs to a protein subfamily containing a tryptophan-aspartic acid-domain (WD) repeat structure. Compelling evidence indicates that RACK1 can interact with many signal molecules and affect different signal transduction pathways. In this study, we cloned a maize *RACK1* gene (*ZmRACK1*) by RT-PCR. The amino acid sequence of ZmRACK1 had seven WD repeats in which there were typical GH (glycine-histidine) and WD dipeptides. Comparison with OsRACK1 from rice revealed 89% identity at the amino acid level. Expression pattern analysis by RT-PCR showed that *ZmRACK1* was expressed in all analyzed tissues of maize and that its transcription in leaves was induced by abscisic acid and jasmonate at a high concentration. Overexpression of *ZmRACK1* in maize led to a reduction in symptoms caused by *Exserohilum turcicum* (Pass.) on maize leaves. The expression levels of the pathogenesis-related protein genes, *PR-1* and *PR-5*, increased 2.5–3 times in transgenic maize, and reactive oxygen species production was more active than in the wild-type. Yeast two-hybrid assays showed that ZmRACK1 could interact with RAC1, RAR1 and SGT1. This study and previous work leads us to believe that ZmRACK1 may form a complex with regulators of plant disease resistance to coordinate maize reactions to pathogens.

## 1. Introduction

RACKl (receptor for activated C kinase 1) is a member of the tryptophan-aspartate repeat (WD-repeat) protein family that was originally identified as a receptor of the active form of protein kinase C (aPKC). It is composed of seven WD40 motifs, which are predicted to form a seven-bladed propeller structure. RACKl was first isolated from chicken liver cells and a human B-lymphoblastoid cell line (B-LCL) [1]. The first RACK1 homologue in plants was identified in tobacco BY-2 suspension cells as an auxin-inducible gene, *arcA* [2]. Subsequently, amino acid sequence homologues of RACK1 were found in all eukaryotes examined (reviewed by Xu and Min [3]). Unlike other organisms having a single *RACK1* gene, RACK1 is encoded by a gene family in some plants. The *Arabidopsis* genome contains three *RACK1* genes, and at least two copies of the *RACK1* homologous exist in the rice genome [4].

RACK1 was found to be a core component of the eukaryotic 40S ribosomal subunit in yeast [5], fungi [6], algae [7], mammals [8] and plants [9] and plays an essential role in fundamental cellular activities, such as transcription and translation, as well as cell proliferation. As a scaffold protein, RACK1 has been reported to interact with more than eighty diverse proteins in metazoans, mediating diverse signaling pathways, which range from cell cycle control [10] to proteasome degradation [11]. RACK1 is also required for various developmental stages in *Xenopus* [12], *Drosophila* [13], *Trypanosoma bruce*i [14] and some fungi [15].

Accumulating evidence suggests that plant RACK1s may also play a regulatory role in multiple developmental processes. In *Arabidopsis*, loss-of-function mutations in RACK1A confer defects in seed germination, leaf production and flowering. Analyses of *rack1* double and triple mutants showed that *rack1b* and *rack1c* can strengthen the *rack1a* mutant’s developmental defects, and an excessive developmental defect and lethality were observed in the triple mutant [16]. The *rack1a* mutants displayed reduced sensitivity to GA (gibberellin) and brassinosteroids, but sensitivity to ABA (abscisic acid) was increased [4]. Downregulation of *RACK1* gene expression by RNA interference enhances drought tolerance in rice [17]. The RACK1 of *Phaseolus vulgaris* has a pivotal role in cell expansion and in symbiosome and bacteroid integrity during nodule development [18].

Many reports have indicated that RACK1 is associated with the immune system and diseases in mammals. Alterations in RACK1 homeostasis resulted in various aspects of disease (reviewed by Adams *et al*. [19]). Nakashima *et al*. [20] demonstrated that RACK1 proteins are involved in the response to pathogens in plants. Overexpression of *RACK1* in rice leads to a reduction in symptoms caused by *Magnaporthe grisea*, reactive oxygen species (ROS) and the induction of pathogenesis-related (PR) genes. Furthermore, RACK1A was shown to interact with multiple proteins in the Rac1 (a ROP/RAC small GTPase) immune complex.

Thus, it appears that RACK1 plays an essential role in multiple developmental processes and innate immunity in plants. However, RACK1 proteins show distinct functions between *Arabidopsis* and rice. Therefore, characterization of the homologs of RACK1 in various plants is necessary. In this work, we isolated a *RACK1* gene from maize encoding a protein sequence showing 89% identity to OsRACK1 from rice. We describe here the characterization of maize RACK1 and its function in disease resistance.

## 2. Results and Discussion

### 2.1. Cloning and Bioinformatic Analysis of ZmRACK1

The *ZmRACK1* cDNA isolated from *Zea mays* was 1005 bp in length, consisting of a single open reading frame. The ORF encoded a polypeptide of 334 amino acids with a calculated molecular mass of 36.2 kDa and a pI of 6.59. The amino acid sequence of ZmRACK1 had seven WD (tryptophan-aspartic acid-domain) repeats in which there were typical GH (glycine-histidine) and WD dipeptides and two internal sequences (Figure S1) that represent the conserved activated protein kinase C (aPKC) binding domains. Comparison of ZmRACK1 with other reported similar sequences revealed that the closest matches were *Oryza sativa* RACK1 with 89% identity, arcA of *N. tabacum* (75%) and ARATH3 of *A. thaliana* (73%). A lower significant identity (65%) was found with human RACK1. The Maize GDB BLAST (http://blast.maizegdb.org/home.php?a=BLAST_UI) results indicated that two related sequences of the *RACK1* gene are present in the genome of *Zea mays* inbred line B73. They share 98.8% amino acid identity (data not shown). The Gene ID of the *ZmRACK1* that we isolated is GRMZM2G038032, which is on chromosome 6, and its homologous GRMZM2G04077 is on chromosome 8.

### 2.2. Expression Pattern of ZmRACK1

**Figure 1:**
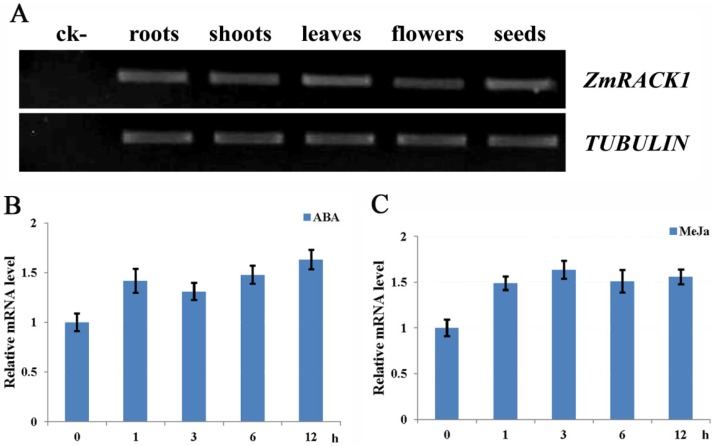
Expression analysis of *ZmRACK1*. (**A**) Semi-quantitative RT-PCR analysis of total RNA prepared from different tissues of *Zea mays*. The *TUBULIN* gene was used as an internal control. The total RNA from leaves without reverse transcription was used as a negative control; (**B**) *ZmRACK1* mRNA expression induced by ABA; and (**C**) *ZmRACK1* mRNA expression induced by methyl jasmonate (MeJa). The transcript levels for *ZmRACK1* were measured by real-time PCR in wild-type maize plants treated with 100 μM of ABA or 10 μM of MeJa. Real-time PCR data were normalized to the tubulin transcript and untreated plants as controls. The experiment was repeated three times, with RT-PCR reactions repeated three times independently. Bars represent means ± SD of three biological replicates.

To analyze the expression pattern of *ZmRACK1*, RT-PCR was performed with total RNA samples extracted from different maize tissues. As illustrated in Figure 1A, The *ZmRACK1* transcript was accumulated in all of the analyzed tissues, including roots, shoots, leaves, flowers and seeds. This result is in accordance with the fact that RACK1 is highly expressed in most tissues of animals [21]. As an essential regulator of signaling pathways in many key biological processes, over 80 binding partners for RACK1 have been reported to date. Its relatively constant expression level implies that RACK1 probable engages in different sets of signaling pathways in different cells though differential expression of its binding partners [19].

Accumulating evidence suggests that plant RACK1s may be involved in hormone responses. The *RACK1* genes from tobacco and alfalfa were induced by auxin and cytokinin, respectively [2,22]. In *Arabidopsis*, *rack1a* mutants were hypersensitivity to ABA (abscisic acid) during seed germination and early seedling growth and less sensitive to auxin during root formation. Furthermore, *rack1a* mutants showed reduced sensitivity to GA (gibberellin) and brassinosteroids during seed germination [23]. Rice *RACK1A* expression was induced by methyl jasmonate (MeJa), ABA and IAA (indole-3-acetic acid) [20]. Considering its shared structural features with OsRACK1, we speculated that ZmRACK1 might also respond to ABA and MeJa. To verify this hypothesis, we grew wild-type *Z. mays* seedlings under 100 μM of ABA conditions and detected *ZmRACK1* transcripts using real-time PCR. The MeJa treatment was performed by spraying the expanding leaves with 10 μM of MeJa. Induction of ZmRACK1 expression was observed in maize leaves after treatment with ABA and MeJa. In comparison with untreated leaves, the expression level increased 1.5 times after 1 h of ABA or MeJa treatment and showed no distinct difference with increased treatment time. Only a slight difference was observed between ABA and MeJa treatments (Figure 1B,C). Nakashima *et al*. [20] found that RACK1 expression improved in wild-type rice suspension cells after 1 h treatment with ABA and MeJa, but the expression level was not increased more than two times in the following 9 h of continuous treatment. *ZmRACK1* had a similar expression pattern to rice *RACK1*, suggesting that they may have similar functions.

### 2.3. Intracellular Localization of ZmRACK1

**Figure 2:**
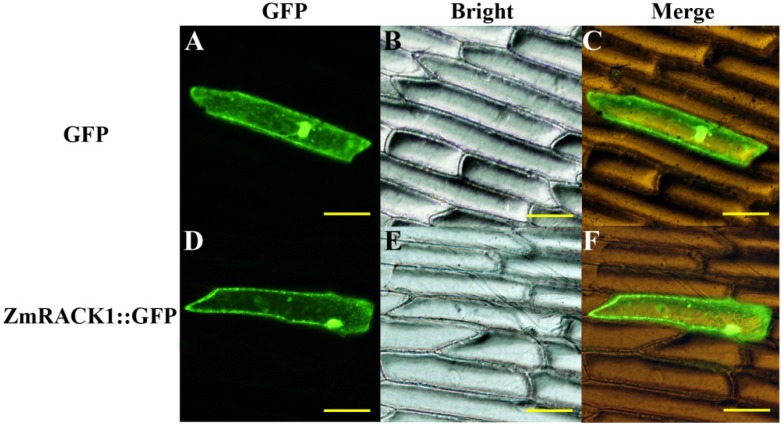
Subcellular localization of ZmRACK1::GFP fusion proteins. GFP alone (**A**–**C**) and ZmRACK1::GFP (**D**–**F**) driven by the CaMV 35S promoter were transformed into onion epidermal cells. Scale bar = 25 μm.

The RACK1 sequence does not contain any known organelle localization motifs. In mammalian cells, RACK1 localized in the cytosol, nucleus and the endoplasmic reticulum [19]. In rice, RACK1A protein was detected in both cytosolic and microsomal fractions [20]. To determine the intracellular localization of ZmRACK1, an expression vector containing ZmRACK1 fused with GFP was constructed and transformed into onion epidermal cells by particle bombardment. Fluorescence visualization showed GFP fluorescence in the cytoplasm, nucleus and plasma membrane of the cells (Figure 2). As a ribosome-associated protein, RACK1 has a key role in the translation machinery. It has been reported that RACK1 interacts with *Arabidopsis* eukaryotic initiation factor 6 (eIF6), a key regulator of the 80S ribosome assembly [24]. Moreover, RACK1 has been identified in several complexes in the nucleus, leading to the transcription of several genes [25,26]. In addition to anchor proteins at particular locations, RACK1 has a significant role in shuttling proteins around the cell. Adams *et al*. [19] suggested that the translocation of RACK1 among various cellular compartments is most likely influenced by the particular cohort of proteins interacting with RACK1 at any one time, rather than being controlled by posttranslational modification of RACK1 itself.

### 2.4. Overexpression of ZmRACK1 in Maize Confers Resistance to Exserohilum turcicum

A previous work suggested that RACK1 not only plays a critical role in plant development, but also participates in innate immunity and response to abiotic stress [20]. To investigate the function of the *ZmRACK1* gene, plant expression vectors with sense *ZmRACK1* and RNAi driven by the maize ubiquitin promoter were introduced into maize embryonic callus by particle bombardment. Transformed plants resistant to hygromycin were obtained, and genomic DNA PCR analysis was subsequently carried out on nine transformed lines with the sense gene and six transformed lines with the RNAi vector. The stability of transgene inheritance in the T1 progenies was checked by PCR analysis. The transgene was detected in the T1 progenies of all transgenic lines, and segregation of the transgene was in accordance with Mendel’s law in four overexpressing lines (Table 1). These results indicated that the *ZmRACK1* transgene was integrated as a monolocus into the maize genome and inherited by the T1 progenies.

**Table 1:** PCR analysis of T1 transgenic plants.

Vector	Line	Plant number of PCR positive	Plant number of PCR negative
pRHU-RACK1	36	12	4
	37	12	3
	38	14	4
	39	11	3
pRHU-RACK1i	40	8	7
	41	11	3
	42	7	5

**Figure 3:**
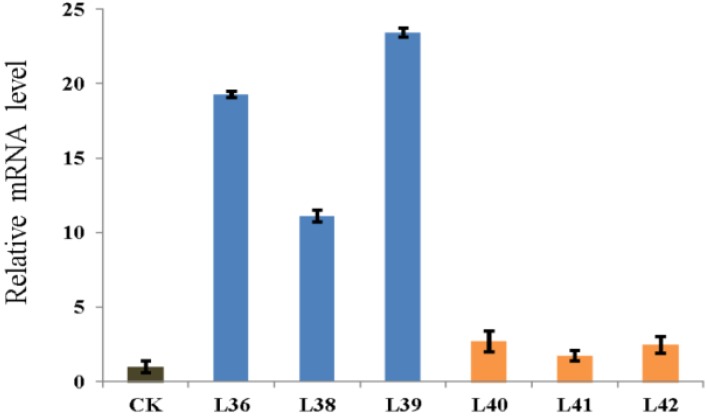
Quantitative real-time RT-PCR analysis of the *ZmRACK1* gene in the leaves of transformed maize. CK, wild-type plants. L36, L38 and L39, overexpression plants. L40, L41 and L42, RNAi plants. Bars represent means ± SD of three biological replicates.

The expression of *ZmRACK1* in T2 transgenic plants was analyzed by real-time PCR. As shown in Figure 3, transgenic plants, L36, L38 and L39, harboring Ubi1-ZmRACK1, showed higher accumulation of *RACK1* transcripts in seedlings compared with the control plant, while *ZmRACK1* transcripts were still detectable in the three transgenic lines carrying the RNAi vector. These results indicated that the *ZmRACK1*-overexpression construct worked effectively, but the RNAi vector did not.

Recent evidence showed that transgenic rice plants overexpressing *RACK1A* had increased resistance to a compatible race of rice blast fungus [20]. Considering that *ZmRACK1* shared the same response to ABA and MeJa with OsRACK1, we speculated that the function of ZmRACK1 in signaling pathways was similar to that of OsRACK1. Therefore, the disease resistance of transgenic maize plants overexpressing *ZmRACK1* was examined.

**Figure 4:**
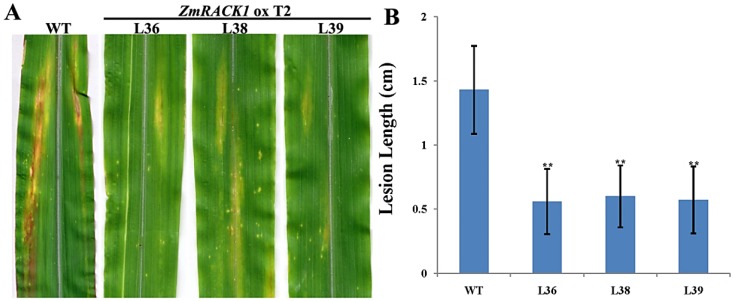
Effect of *ZmRACK1* overexpression in maize. (**A**) *ZmRACK1*-overexpressing maize leaves (L36, L38 and L39) and non-transgenic maize leaves (WT) after infection with *E. turcicum*. ox T2 represents T2 progenies of overexpression lines; (**B**) Quantitative analysis of the increased resistance of transgenic plants to infection with *E. turcicum*. The results represent means. Asterisks indicate statistically significant differences between the WT and overexpression plants (paired *t-*test, ** *p* < 0.01).

Northern corn leaf blight of maize, caused by *Exserohilum turcicum* (Pass.), is a serious disease that causes yield loss, nutritive value alteration, lowered germinative capacity of the seeds and stalk rot [27]. T2 homozygous monolocus lines overexpressing *ZmRACK1* were evaluated for resistance against *E. turcicum*. A conidial suspension of *E. turcicum* was sprayed on the leaf surfaces of transgenic maize when they reached the three-leaf stage. Foliar infection was measured 14 days after infection of 16 plants for each line. The size of chlorotic and necrotic lesions and the rate of lesion expansion were observed to evaluate the resistance to *E. turcicum*. Results were obtained from two independent experiments performed on three lines (L36, L38 and L39). Lesions began to appear three days after incubation on the three overexpressing lines, L36, L38 and L39, and the wild-type; these lesions were yellow to gray and expanded from the vein to the leaf edge. The transgenic plants exhibited a significant decrease in disease symptoms compared with the WT line. A significant difference was found in the size of the chlorotic and necrotic lesions and the rate of lesion expression. Visual observations on Lines L36, L38 and L39 revealed a pronounced decrease in leaf yellowing and in the area of necrosis (Figure 4A). The lesion length in *ZmRACK1*-overexpressing plants ranged from 0.33 to 0.89 cm. Statistical analysis indicated that the lesion length reduction in all three transgenic lines was highly significant (paired *t*-test, *p* < 0.01, Figure 4B), suggesting that ZmRACK1 overexpression enhanced maize resistance against the leaf blight pathogen, *E. turcicum*.

### 2.5. ZmRACK1 Regulates ROS Production and PR Gene Expression

**Figure 5:**
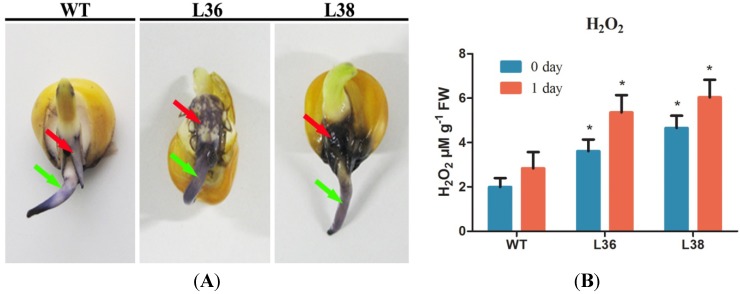
ROS production in T2 transgenic maize lines overexpressing *ZmRACK1*. (**A**) NBT (nitroblue tetrazolium) staining of ROS production in germinated seeds. Maize seeds were germinated for 5 days and stained with 0.1% nitroblue tetrazolium (NBT) to visualize ROS production. Red and green arrows indicate the sites of increased ROS production; and (**B**) Production of H_2_O_2_ in leaves after infection with *E. turcicum*. The level of H_2_O_2_ was measured using leaves before and one day after *E. turcicum* inoculation. The data represent the results of two independent experiments (*n* = 3). FW, fresh weight. WT, wild-type maize as a control. Asterisks indicate statistically significant differences between the WT and overexpression plants (paired *t*-test, * *p* < 0.05).

When a pathogen attacks, a plant’s innate immunity displays innate pathogen-specific resistance by producing reactive oxygen species (ROS) and phytoalexins, a change of cell wall composition and inducing the expression of pathogenesis-related genes. ROS have been known to be messengers in cell signal transduction and cell cycling and to play a key role in plant defense responses to pathogen infection [28]. To analyze ROS production in transgenic maize plants, after five days of germination, the seeds were stained with 0.1% NBT (nitroblue tetrazolium). The results showed that ROS were produced from both overexpression lines and the wild-type, but a darker color was observed in the corresponding position of the transgenic lines (Figure 5A). Quantitative analysis also showed that H_2_O_2_ was increased in the leaves overexpressing *ZmRACK1* (Figure 5B). We also examined the response to the infection with *E. turcicum* of transgenic plants. Leaves of *ZmRACK1*-overxpressing plants showed significantly higher H_2_O_2_ production than the non-transgenic plants one day after *E. turcicum* inoculation (Figure 5B). These results indicate that ZmRACK1 is related to ROS production in maize.

Pathogenesis-related (PR) proteins are a group of heterogeneous proteins that are induced by pathogenic infections and by related abiotic stresses. Production of PR proteins can increase the resistance of the plant against pathogen attack [29]. PR-1 proteins are involved in salicylic acid (SA) active defense responses, which have antifungal activity at the micromolar level against a number of plant pathogenic fungi [30]. PR-5 proteins are involved in acquired systemic resistance and responses to biotic stress [31]. It has been reported that PR-1 and PR-5 can act as marker genes in the disease resistance process of maize [32].

**Figure 6:**
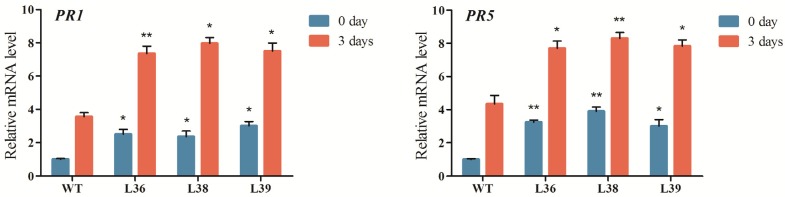
Expression analysis of the pathogenesis-related genes, *PR1* and *PR5*, in *ZmRACK1*-overexpressing maize by real-time RT-PCR. The transcript levels of *PR1* and *PR5* increased in the three *ZmRACK1*-overexpressing lines, L36, L38 and L39, before and three days after *E. turcicum* inoculation. Bars represent the means ± SD of three biological replicates. Asterisks indicate statistically significant differences between the WT and overexpression plants (paired *t-*test, * *p* < 0.05, ** *p* < 0.01).

To determine whether disease resistance in transgenic plants was accompanied by the expression of *PR* genes, leaves from the wild-type and transgenic Lines L36, L38 and L39 were collected before and three days after *E. turcicum* inoculation and used for RNA extraction. Real-time PCR analysis showed that *PR-1* and *PR-5* were induced to a high level in the transgenic plants under normal conditions. The transcription levels of *PR-1* and *PR-5* were 2.5–3 times greater in Lines L36, L38 and L39 than in the wild-type (Figure 6). The accumulations of *PR-1* and *PR-5* gene mRNAs were increased significantly in wild-type and transgenic plants after *E. turcicum* inoculation. In comparison with the wild-type, the expression level increased more than two times in the three *ZmRACK1*-overexpressing lines three days after *E. turcicum* inoculation. These results suggest that overexpression of *ZmRACK1* activates the transcription of pathogenesis-related genes in transgenic maize, therefore improving resistance to *E. turcicum* infection.

### 2.6. ZmRACK1 Interacts with RAC1, RAR1 and SGT1

ZmRACK1 confers resistance to the maize pathogen, *E. turcicum*, possibly through the interaction with pathogenesis-related proteins. It has been reported that Rac1 is a regulator of reactive oxygen species (ROS) production, and it also activates pathogenesis-related gene expression in rice [33]. Thao *et al*. [34] speculated that these effects were mediated by Rac1 interaction with other regulators of plant disease resistance, such as RAR1 and SGT1, to form an immune complex. RACK1 has been proven to be a member of the immune complex, functioning as molecular glue, binding Rac1 together with other interaction proteins in rice [20].

**Figure 7:**
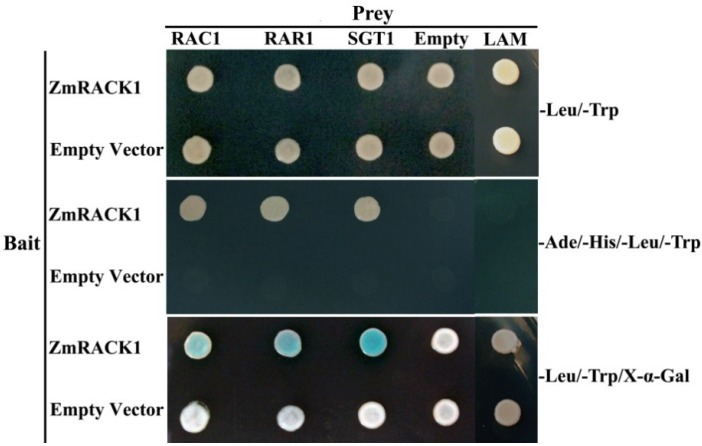
Interaction of ZmRACK1 with RAC1, RAR1 and SGT1 in yeast two-hybrid assays. The different plasmid combinations co-transformed into yeast cells are indicated at the left and the top. Survival of the cells in SD (synthetic defined) medium without tryptophan, leucine, histidine and adenine (SD-TLHA) and blue colonies indicated an interaction between the two co-transformed gene products. An irrelevant prey protein, LAM (lamin C), was used as a negative control.

To confirm whether ZmRACK1 is also involved in maize innate immunity, the interaction of ZmRACK1 with RAC1, RAR1 and SGT1 was tested by a yeast two-hybrid assay. After co-transformation, the yeast growth in SD (synthetic defined) medium lacking tryptophan and leucine was monitored to ensure that there were no growth defects in the recombinant clones. Specific interactions were confirmed by analysis of the ability of clones that harbored the binding proteins to survive in SD selective medium lacking tryptophan, leucine, histidine and adenine (SD-TLHA) and to induce β-galactosidase. The results showed that ZmRACK1 interacted with RAC1, RAR1 and SGT1 (Figure 7). RAR1 (required for *Mla12* resistance) is an important component of R gene-mediated disease resistance and functions upstream of H_2_O_2_ accumulation in attacked host cells [35]. SGT1 (for the suppressor of the G2 allele of *skp1*) is required for disease resistance mediated by diverse R proteins [36]. As a scaffold protein, ZmRACK1 can interact with Rac1, RAR1 and SGT1 directly, indicating that ZmRACK1 is a key element of the signaling complex in the regulation of the immune response in maize.

## 3. Experimental Section

### 3.1. Plant Materials and Fungal Strain

*Z. mays* L. cv. NongDa108 was provided by Professor Qifeng Xu (China Agriculture University, Beijing, China). *Z. mays* L. inbred lines Zong31 and Qi31 were given to us by Professor Jingrui Dai (China Agriculture University, Beijing, China). The fungal strain, *Exserohilum turcicum*, was provided by Jingao Dong (Hebei Agriculture University, Baoding, China).

### 3.2. Cloning of the ZmRACK1 Gene

Total RNA was isolated from *Z. mays* cv. NongDa108 seedlings by the TRIzol method, according to the instructions (Invitrogen, Carlsbad, CA, USA) and treated with DNase I (TaKaRa, Dalian, China). This RNA was used as a template, and cDNA was synthesized using reverse transcriptase followed by PCR amplification. A sense primer, RK1, and an antisense primer, RK2 (Table S1), were designed based on the nucleotide sequence of the *Z. mays* mRNA sequence (GenBank: BT016269). Amplification conditions were 95 °C pre-denaturation for 5 min, 30 cycles of 95 °C for 1 min, 51 °C for 1 min, 72 °C for 1 min and then 10 min at 72 °C. The PCR products were recovered from a 0.8% agarose gel. The purified fragment was cloned into the pMD 18-T vector (TaKaRa, Dalian, China), and the nucleotide sequence of the cDNA insert was determined by sequencing (Sangon, Shanghai, China).

### 3.3. Treatment of Seedlings with MeJA and ABA

Maize seeds were germinated on water-saturated filter paper at 27 °C in the dark after surface sterilization with 0.5% (*v*/*v*) hypochlorite for 20 min. Germinated seedlings were grown on soil:vermiculite (3:1, *v*/*v*) at 27 °C in an environment-controlled chamber under a white fluorescent lamp with a 16/8 h light/dark photoperiod and 75% relative humidity. When the second leaf was fully expanded, ABA treatment was carried out by transferring the maize seedlings to Hoagland’s nutrient solution containing 100 μM ABA (Sigma, Madison, WI, USA) for 1, 3, 6 or 12 h. For the MeJA treatment, seedlings with three leaves were sprayed with 10 μM MeJA (Sigma, Madison, WI, USA). After treatment, the second fully-expanded leaves were picked for total RNA extraction and RT-PCR. Three biological replicates were performed per treatment with six plants.

### 3.4. Real-Time PCR

The leaves of regenerated maize plants harboring the pRHU-RACK1 or pRHU-RACK1i vector and the wild-type were collected for RNA isolation after treatment with MeJA and ABA. cDNA was synthesized from total RNA after treatment with DNase I and used for quantitative analysis of gene expression using the SYBR Green PCR master mix (GenStar, Beijing, China) with the gene-specific primers, Rkr5 and Rkr3 (Table S1). The *α-tubulin* gene was used as an internal control and was amplified with the primers, Tu5 and Tu3 (Table S1). Data were collected using the ABI PRISM 7000 (Invitrogen, Carlsbad, CA, USA) sequence detection system according to the instruction manual.

For the examination of pathogenesis-related genes, RNA was isolated from the leaves of young T2 plants before and 3 days after infection with *Exserohilum turcicum* and cDNA was synthesized as described above. Primers PR1-5 and PR1-3 (Table S1) were used for the *PR-1* gene. For *PR-5*, the primers, PR5-5 and PR5-3 (Table S1), were used.

### 3.5. Transient Expression in Onion Epidermis Cells

The GFP (green fluorescence protein) fragment amplified from pTACgfp (provided by professor Guoqin Liu, China Agriculture University, Beijing, China) using the primers, pG1 and pG2 (Table S1), was fused to the *C*-terminus of ZmRACK1. Expression of the GFP control and ZmRACK1-GFP was driven by the CaMV35S promoter. The onion inner epidermis was spread on the center of an MS plate and bombarded with 1100 psi of helium at a 7 cm distance with a PDS-1000/He (Bio-Rad, Hercules, CA, USA). After bombardment, the onion epidermal cells were incubated at 25 °C without light for 24 h and then observed under a fluorescence microscope (Olympus BX51, Tokyo, Japan). The excitation wavelength was 488 nm for fluorescein isothiocyanate excitation, and emission filters were 505/530 nm for GFP fluorescence.

### 3.6. Plasmid Constructs and Maize Transformation

To produce *ZmRACK1* overexpression and RNAi vectors, the intermediate vector, pRHU, was constructed first. The CaMV35S promoter in pROK219 was replaced with the maize ubiquitin promoter (Ubi1) from pAHC25 and a cassette with a CaMV35S promoter, a hygromycin resistance gene and a nopaline synthase terminator from pHyg was inserted into the *Hin*dIII site to obtain pRHU. The overexpression construct, pRHU-RACK1, was generated by subcloning the *RACK1 Kpn*I and *Sac*I digested fragments and ligating them into the corresponding sites of pRHU under control of the Ubi1 promoter. For the *ZmRACK1* RNAi construct, by excising, filling in and ligating into the vector, pBluescript SK(+), a fragment with the *ZmRACK1* gene in the sense and antisense orientation was ligated to GUS. This ZmRACK1s-gus-ZmRACK1an fragment was excised with *Sac*I and *Kpn*I and inserted into the corresponding sites of the vector, pRHU, to yield pRHU-RACK1i.

The plasmids, pRHU-RACK1 and pRHU-RACK1i, were introduced into embryogenic callus from the maize hybrid Zong31 × Qi31 by microprojectile bombardment. After regeneration on selective medium, transformed maize lines were detected for the presence of the transgene by PCR.

### 3.7. Infection of Maize Plants with Exserohilum turcicum

The seeds of T2 transgenic maize were planted in pots containing vermiculite and grown in the greenhouse at 25–28 °C and a relative humidity of 30%–60% under natural daylight until the 3- to 4-leaf stage. 

*Exserohilum turcicum* cultures were grown on OA (Oatmeal agar) medium with 18 g/L glucose at 28 °C in dark for 10 days and then transferred to 20 °C under a 12/12 h light/dark photoperiod for 3 days. A conidial suspension was prepared by flooding the cultures with sterile distilled water, scraping the surface with microscope slides to dislodge the conidia and then filtering using cheese cloth. The concentration of the conidial suspension was adjusted to 2 × 10^4^ conidia per mL using a hemocytometer. Tween 20 was added at a final concentration of 0.1% to assist in the dispersion of the spores. The conidial suspension was sprayed onto the leaf surfaces at equidistant points. Incubation was performed in moist conditions (90% relative humidity) at 23 °C in growth chambers illuminated for 12 h per day. Photographs of disease lesions were taken 2 weeks after inoculation. The general size ranges of the necrotic and chlorotic zones of the lesions were measured with a ruler. Three leaves of each plant were measured, and three biological replicates were performed with six plants.

### 3.8. Detection of ROS

The seeds of T2 transgenic maize were germinated on water-saturated filter paper at 27 °C in the dark. Superoxides generated during the seedling stage were detected by immersing the seedlings in 0.1% (*w*/*v*) nitroblue tetrazolium [20].

### 3.9. Quantitation of H_2_O_2_

The H_2_O_2_ leve1s in plant tissues were measured following the method described by Patterson *et al*. [37] with some modification. Leaf tissue from each plant before and 1 day after infection with *Exserohilum turcicum* were collected and homogenized in ice-cold acetone at a ratio of 1.0 g sample to 2 mL acetone. The crude homogenate was centrifuged at 3000× *g* for 10 min. Titanium reagent (0.1 mL 20% TiCl_2_ in concentrated HCl) was added to 1 mL extract supernatant and then precipitated by adding 0.2 mL 17 M ammonia solution. The precipitate was isolated by centrifugation and then washed five times with ice-cold acetone. Finally, the precipitate was dissolved in 3 mL 1 M H_2_SO_4_, and the absorbance of the solution was measured at 410 nm using a UV2300 spectrophotometer (Techcomp, Shanghai, China). Three biological replicates were performed.

### 3.10. Yeast Two-Hybrid Constructs and Methods 

Yeast two-hybrid screens were performed with the Clontech Matchmaker GAL4 Two-Hybrid System 3 (Clontech, Palo Alto, CA, USA). A bait vector carrying ZmRACK1 was produced by cloning *ZmRACK1* into pGBK-T7. The coding regions of the *RAC1*, *RAR1* and *SGT1* genes of maize were amplified using the primers, RAC5/RAC3, RAR5/RAR3 and SGT5/SGT3 (Table S1), respectively. These primers were designed based on the nucleotide sequences of the *Z. mays* mRNAs in GenBank: NM_001111460 (*RAC1*), NM_001159033 (*RAR1*) and NM_001155651 (*SGT1*). It is known that Rac1 is localized to the plasma membrane by the Cys in its *C*-terminus [38]. Therefore, site-specific mutagenesis of the *C*-terminal Cys was performed by PCR using the primers, mRAC5 and mRAC3 (Table S1), resulting in Ser codons replacing the Cys codons to ensure that Rac1 was transferred into the nucleus. The resultant gene fragments were inserted into the prey vector, pGAD-T7. All plasmid constructs were verified by sequencing. Combinations of bait and prey vectors were introduced into the cells of *Saccharomyces cerevisiae* strain AH109. A positive interaction between two proteins was indicated by the growth of yeast colonies on SD selective medium lacking tryptophan, leucine, histidine and adenine (SD-TLHA). The transformations were confirmed by PCR. The positive clones were also assayed by a filter lift α-galactosidase assay for LacZ activity, as described in the manufacturer’s protocols.

## 4. Conclusions

RACK1 is a member of the WD-repeat protein family that plays divers roles as a scaffold protein [39]. *ZmRACK1* was isolated from *Z. mays* and encodes a protein with seven WD repeats, including typical GH and WD dipeptides. Amino acid sequence comparison revealed that ZmRACK1 was similar to *Oryza sativa* RACK1, arcA of *N. tabacum* and ARATH3 of *A. thaliana*.

As a multi-functional scaffold protein, RACK1 is associated with different signal transduction pathways [40] and also binds with membrane acceptors and transcriptional factors [41]. In addition, as a ribosome-binding protein, RACK1 has an effect on protein translation [42]. These processes are not independent; they form a three-dimensional network to regulate some of the central life processes in diverse organisms. Recent studies have shown that *Arabidopsis* RACK1 is a negative regulator of ABA responses. ABA influenced the interaction between RACK1 and eIF6, and this conserved interaction likely mediates ribosome assembly. Therefore, the translation of proteins required for seed germination was repressed, and seed germination was inhibited eventually [23,24]. However, the expression of RACK1 in rice cells is upregulated by ABA [43]. Rice RACK1A expression is also induced by methyl jasmonate and IAA [20]. This inconsistency between RACK1s from rice and *Arabidopsis* in response to ABA indicates that they may have distinct functions. In this study, we found that ZmRACK1 shared similar ABA and MeJa responses with OsRACK1. Therefore, we speculate that the function of ZmRACK1 might be more or less similar to that of OsRACK1.

Scaffolding proteins simultaneously associate with several partners to form signaling complexes, thus enhancing the specificity and efficiency of signal transduction. In this study, we found that ZmRACK1 regulates ROS production, and yeast two-hybrid assays showed that it can interact with RAC1, RAR1 and SGT1. Furthermore, overexpression of *ZmRACK1* in maize led to a reduction in symptoms caused by *E. turcicum* on maize leaves. The expression levels of the pathogenesis-related protein genes *PR-1* and *PR-5* increased 2.5–3 times in transgenic maize, and ROS production was more active than in the wild-type. Based on these results, we conclude that ZmRACK1 may form a complex with regulators of plant disease resistance to coordinate maize reactions to pathogens. However, the functional mechanism of RACK1 in maize innate immune responses needs to be further studied.

In addition, several studies have shown that RACK1 plays important roles in plant responses to environmental stress. Using RNA interference technology, Li *et al*. [17] obtained transgenic rice in which the *RACK1* gene expression level was inhibited by around 50%. Compared with non-transgenic rice, the RNAi plants showed higher tolerance to drought stress. The peroxidation of membranes and the production of malondialdehyde were significantly reduced, while superoxide dismutase activity was significantly higher than in non-transgenic rice plants. This suggested that RACKl negatively regulated rice tolerance to drought stress. However, Nakashima *et al*. [20] could not obtain regenerated rice plants from transformed RACK1A-RNAi calli. Similarly, in this study, we failed to get transgenic maize plants in which *ZmRACK1* expression was inhibited, so no related information was obtained. We analyzed proline content, a drought resistance-related indicator, of *ZmRACK1* overexpression plants, and found that there was no significant difference compared with wild-type corn (data not shown). Therefore, whether ZmRACK1 is involved in drought stress responses needs to be further researched.

RACK1, a functionally diverse scaffold protein in many organisms, has been much studied in animals, but research in plants has been relatively limited. Further investigation is required to understand the functions of plant RACK1s.

## Supplementary Files

Supplementary File 1 Supplementary Information (PDF, 569 KB)
