# Supplementary material for: Maize ZmRACK1 Is Involved in the Plant Response to Fungal Phytopathogens
Source: Int J Mol Sci. 2014 May 26;15(6):9343–59. doi: 10.3390/ijms15069343 (PMC4100098; doi:10.3390/ijms15069343)
Supplement: Supplementary File 1 — Supplementary Information (PDF, 569 KB) [file ijms-15-09343-s001.pdf]

Supplementary Information

Figure S1. Multiple amino acid sequence alignment of some WD40-repeat proteins. Amino acids that are identical or similar are shaded in black or gray, respectively. Gaps are shown as dashed lines. The proteins aligned are ArcA (*Nicotiana tabacum*, D17526), AtArcA (*Arabidopsis thaliana*, U77381), RACK1 (*Homo sapiens*, BC035460), OsRACK1 (*Oryza sativa*, D38231) and ZmRACK1 (*Zea mays*). The positions of GH and WD dipeptides in each WD40 repeat are indicated by triangles and asterisks, respectively, below the residues. Two internal sequences representing the PKC binding domains are underlined. The amino acid sequences were aligned using the CLUSTALW multiple alignment tool of the BioEdit Sequence Alignment Editor: http://www.mbio.ncsu.edu/BioEdit/bioedit.html.

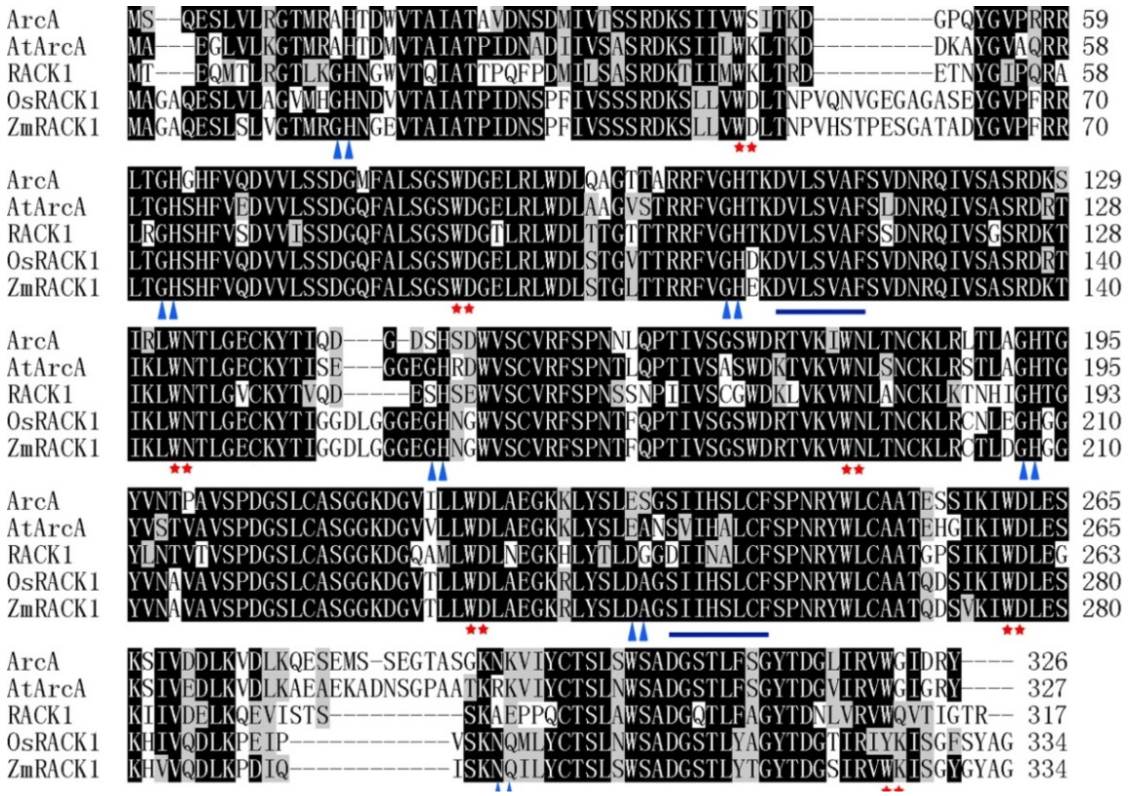

Table S1. Oligonucleotides primers used for various gene constructs and PCR.

**Oligo names primer sequences**		**Features**
Primers for *ZmRACK1*		
RK1	5'-AATCCCTCACAGCAACCAT-3'	
RK2	5'-TCGACATCTAGCCTGCGTA-3'	
RKr5	5'- GATGGATCGCTCTGCGCC-3'	For real-time PCR
RKr3	5'-TAGCGGTTGGGCGAGAAGC-3'	For real-time PCR
Primers for maize *tubulin*		
Tu5	5'-AACTGGGACGATATGGAGAA-3'	For real-time PCR
Tu3	5'-CACTGGCGTATAGGGACAAC-3'	For real-time PCR
Primers for pathogenesis-related genes of maize		
PR1-5	5'-CCTGGGTGTCCGAGAAGCA-3'	For real-time PCR
PR1-3	5'-ACAGCCGATGGCGGTGGAGT-3'	For real-time PCR
PR5-5	5'-CGGCAGCCAGGACTTCTA-3'	For real-time PCR
PR5-3	5'-GCCACAGGCATGGGTCT-3'	For real-time PCR
Primers used in yeast two hybrid		
RAC5	5'-CGGCAGTGAGAGCGATG-3'	
RAC3	5'-AAGAGTCTGTGCTTTACG-3'	
mRAC5	5'-ATGAGCGCGGCGGCGGCG-3'	
mRAC3	5'-TTACGATGTGAAAGATCCGCTTCCACTGA-3'	
SGT5	5'-TCTCGCCCAAGTCATCGT-3'	
SGT3	5'-ACGGCAAGTAGCACTCAGACAC-3'	
RAR5	5'-ATGTCAACGACGACGGAGGC-3'	
RAR3	5'-TCACACCGCATCAGCATTATG-3'	
Primers for gfp		
pG1	5'-CC<u>CCCGGG</u>ATGAGTAAAGGAGAAGAA-3'	*Sma*I underlined
pG2	5'-GC<u>GAGCTC</u>TTATTTGTATAGTTCATC-3'	*Sac*I underlined

© 2014 by the authors; licensee MDPI, Basel, Switzerland. This article is an open access article distributed under the terms and conditions of the Creative Commons Attribution license (http://creativecommons.org/licenses/by/3.0/).
